# Up-Down Hand Position Switch May Delay the Fatigue of Non-Dominant Hand Position Rescuers and Improve Chest Compression Quality during Cardiopulmonary Resuscitation: A Randomized Crossover Manikin Study

**DOI:** 10.1371/journal.pone.0133483

**Published:** 2015-08-12

**Authors:** Xian-Long Zhou, Lei Li, Cheng Jiang, Bing Xu, Huang-Lei Wang, Dan Xiong, Li-Pin Sheng, Qi-Sheng Yang, Shan Jiang, Peng Xu, Zhi-Qiao Chen, Yan Zhao

**Affiliations:** Emergency Center, Zhongnan Hospital of Wuhan University, 169 Donghu Road, Wuhan, Hubei, 430071, China; Azienda Ospedaliero-Universitaria Careggi, ITALY

## Abstract

Previous studies have shown improved external chest compression (ECC) quality and delayed rescuer fatigue when the dominant hand (DH) was in contact with the sternum. However, many rescuers prefer placing the non-dominant hand (NH) in contact with the sternum during ECC. We aimed to investigate the effects of up-down hand position switch on the quality of ECC and the fatigue of rescuers during cardiopulmonary resuscitation (CPR). After completion of a review of the standard adult basic life support (BLS) course, every candidate performed 10 cycles of single adult CPR twice on an adult manikin with either a constant hand position (CH) or a switched hand position (SH) in random order at 7-day intervals. The rescuers’ general characteristics, hand positions, physiological signs, fatigue appearance and ECC qualities were recorded. Our results showed no significant differences in chest compression quality for the DH position rescuers between the CH and SH sessions (p>0.05, resp.). And also no significant differences were found for Borg score (p = 0.437) or cycle number (p = 0.127) of fatigue appearance after chest compressions between the two sessions. However, for NH position rescuers, the appearance of fatigue was delayed (p = 0.046), with a lower Borg score in the SH session (12.67 ± 2.03) compared to the CH session (13.33 ± 1.95) (p = 0.011). Moreover, the compression depth was significantly greater in the SH session (39.3 ± 7.2 mm) compared to the CH session (36.3 ± 8.1 mm) (p = 0.015). Our data suggest that the up-down hand position switch during CPR may delay the fatigue of non-dominant hand position rescuers and improve the quality of chest compressions.

## Introduction

High-quality cardiopulmonary resuscitation (CPR) is vital for survival after cardiac arrest (CA), and chest compressions are key to the performance of CPR [1]. Several components, such as hand position, position of the victim, and position of the rescuer, can alter the quality of external chest compressions (ECC) [2]. The effects of dominant and non-dominant hand position on ECC quality have also been investigated for both professional health providers and novice rescuers based on previous CPR guidelines [3, 4]. According to those studies, dominant hand position could improve ECC quality for professional health providers, but not novice rescuers. However, our recent manikin study based on current guidelines suggested that using the dominant hand position could improve ECC quality and delay the fatigue of novice rescuers [5]. In that study, we also found that approximately 71% (155/220) of our novice rescuers preferred a non-dominant hand position during CPR [5]. Thus, a constant non-dominant hand position may unintentionally decrease the quality of CPR for those rescuers. We considered that if a single non-dominant hand position rescuer switches the up-down hand position during CPR, the proportion of non-dominant hand-position-CPR would decrease; thus, the rescuer’s fatigue would decrease, and the chest compression quality would increase. To date, no study has specifically investigated the effects of up-down hand position switch during CPR on ECC quality or rescuer fatigue. This manikin study was based on the 2010 American Heart Association (AHA) CPR guidelines and was designed to test our hypothesis.

## Methods and Materials

### Ethic statement

This study was conducted in November, 2014 at Zhongnan Hospital of Wuhan University (Wuhan, Hubei, China) and was approved by the ethics committee of that hospital (No: 2014ZN018). Each participant signed an informed consent form.

### Participants

Fifth and sixth year medical students were recruited to participate in this study. All participants were BLS certified or had taken a basic life support (BLS) course within 12 months prior to enrollment. Participants with underlying diseases such as diabetes, heart disease and hypertension were excluded from this study. In addition, participants with back, shoulder, arm, wrist, hand or finger pain were also excluded. The following characteristics of the participants were recorded: age; sex; height; weight; dominant hand; preferred hand position during CPR; clinical specialty; BLS certification status; heart rate (HR) at rest and after chest compressions; blood pressure, including systolic blood pressure (SBP), diastolic blood pressure (DBP) and mean arterial pressure (MAP) before and after compressions; peripheral capillary oxygen saturation (SpO2); and Borg score [6–8] before and after compressions.

### Study protocol

**Fig 1:**
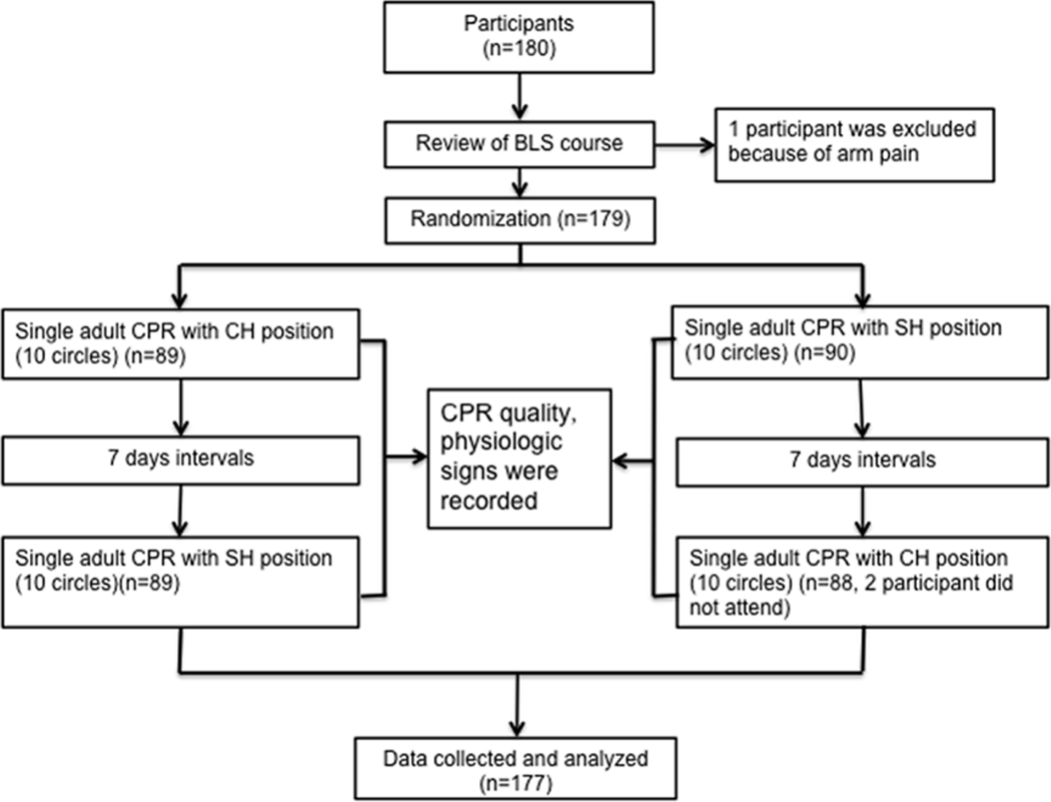
Study protocol. BLS: basic life support; CPR: cardiopulmonary resuscitation; CH: constant hand position; SH: switched hand position.

First, a review of the BLS course based on the 2010 AHA CPR guidelines was conducted. At the end of the review, each participant performed 10 cycles of single adult CPR with either a constant dominant hand position (CH) or up-down switched hand position (SH) after a brief time of practice on an adult Resusci Anne QCPR (Laerdal China Ltd. Hangzhou, China). The hand position of the participants during CPR was assigned randomly using a card in a sealed envelope. If “CH” was on the card in the envelope, 10 cycles of CPR with the constant hand position were performed followed by 10 cycles of CPR with the up-down switched hand position in 7 days. If “SH” appeared on the card, the participants first performed 10 cycles of CPR with the up-down switched hand position and then, another 10 cycles of CPR with the constant hand position in 7 days. The preferred (left or right) hand was constantly in contact with the sternum during the constant hand position session, while for the up-down switched hand position session, chest compression was started with the preferred hand position, and the up-down position of hands was switched every two cycles. For the up-down switched hand group, the two sessions were conducted in random order at 7-day intervals. Two observers supervised the entire procedure to ensure that the participants did not change the hand that was in contact with the sternum during the constant hand position session and that they switched hand position every two cycles during the switched hand position session. For the two sessions, each participant performed chest compressions with the same hand in contact with the sternum for the first cycle. The hand in contact with the sternum during CPR, physiologic signs of the rescuers and CPR quality were noted (Protocol seen in Fig 1).

### Measurements and data collection

An adult Resusci Anne QCPR was used in this study. Single adult CPR was performed according to the 2010 AHA guidelines. A beep sound provided by a speaker guided the participants to switch their hand position every two cycles. A real-time feedback system showed the participants their compression rate and depth and reminded the participants to maintain adequate compression rate and depth as per the guidelines. If the participants believed that fatigue might affect the quality of their chest compressions or they felt great pain or fatigue, they were instructed to inform the investigator immediately, and the observers noted the cycle number of the fatigue appearance. Total number of chest compressions, total time of compressions, number of cycles with correct and incorrect hand placement, number of incomplete chest recoil compressions, compression depth for each chest compression and compression rate during each cycle were recorded. Total hands-off time during CPR, ventilation rate and proportion of adequate ventilations were also recorded.

### Sample size calculation

Sample size was determined using *R* function *power*.*t*.*test* [9]. Pilot experiment led to a rough estimate of the standard deviation of the intra subject SH—CH measurement difference in compression depth equal to 13. Assuming a power of 0.8, and a type I error of 0.05, detecting a difference of 3 mm in compression depth between the two methods would require 150 participants. Finally, we planned to include 20% more, i.e. 180 medical students, anticipating some loss for various reasons.

### Statistical analysis

The data were processed by the statistical analysis software SPSS version 22.0 (SPSS Inc., Chicago, IL). Data were expressed as the means ± SD, percentages or numbers. Paired observations for each participant were analyzed using the paired *Student’s t*-test if the data were normally distributed; otherwise, the non-parametric paired Wilcoxon’s test was used. Percentages were compared using the Chi Square test or Fisher exact examination. The probabilities of event-free (no fatigue appearance) were estimated with the Kaplan-Meier method. The significance of comparison was calculated with the log-rank test. A two-tailed *P* value less than 0.05 was considered significant.

## Results

### Study population demographics

In total, 180 medical students attended a review BLS course and agreed to participate in this study. Two students did not finish the study, and one student was excluded due to arm pain. Thus, 177 participants were included in the study. Table 1 shows the characteristics of all participants. There were 58 (32.8%) dominant hand position participants and 119 (67.2%) non-dominant hand position participants. The mean age of the participants was 24 years (SD ± 2); 71 participants were male (40.1%) and 106 were female (59.9%). The mean weight of the participants was 57 kg (SD ± 10), and the mean height of the participants was 166 (SD ± 8) cm. The mean body mass index (BMI) of the participants was 21 (SD ± 3) kg·m^-2^. The dominant hand of most participants (145/177) was the right hand. Fourteen participants (7.9%) were BLS certified, while the other 163 participants (92.1%) had taken BLS courses within the last 12 months. The disciplines of the students were as follows: internal medicine (55, 31.1%), surgery (37, 20.8%), orthopedics (11, 6.2%), anesthesiology (6, 3.4%), emergency medicine (4, 2.3%), and other departments, such as rehabilitation, clinical laboratory, radiology, etc. (61, 34.5%). The only characteristic that was significantly different between the dominant hand position group and the non-dominant hand position group was weight (DH vs. NH: 59 ± 12 vs. 55 ± 10 kg, p = 0.034) (Table 1).

**Table 1:** Study population demographics.

	All participants (n = 177)	DH participants (n = 58)	NH participants (n = 119)	*p* value ^a^
Gender				
Male, n (%)	71 (40.1)	25 (43.1)	46 (38.7)	0.571
Female, n (%)	106 (59.9)	33 (56.9)	73 (61.3)	
Age, mean (SD) (years)	24 (2.0)	24 (1.5)	24 (2.2)	0.651
Weight, mean (SD) (kg)	57 (10)	59 (12)	55 (10)	0.034
Height, mean (SD) (cm)	166 (8)	166 (8)	165 (8)	0.816
BMI, median (SD) (kg m^-2^)	21 (3)	21 (2)	20 (3)	0.108
Dominant hand				
Right, n (%)	145 (82.5)	48 (82.8)	97 (81.5)	0.840
Left, n (%)	32 (17.5)	10 (17.2)	22 (18.5)	
BLS certified				
Yes, n (%)	14 (7.9)	5 (8.6)	9 (7.6)	0.807
No, n (%)	163 (92.1)	53 (91.4)	110 (92.4)	
Specialties				
Emergency medicine, n (%)	4 (2.3)	1 (1.7)	3 (2.5)	0.758
Anesthesiology, n (%)	6 (3.4)	3 (5.2)	3 (2.5)	
Surgery, n (%)	37 (20.8)	13 (22.4)	24 (20.2)	
Internal medicine, n (%)	55 (31.1)	15 (25.7)	40 (33.6)	
Paediatrics, n (%)	3 (1.7)	2 (3.5)	1 (0.8)	
Orthopedics, n (%)	11 (6.2)	4 (7.0)	7 (5.9)	
Others, n (%)	61(34.5)	20 (34.5)	41 (34.5)	

DH: dominant hand position; NH: non-dominant hand position; SD:standard deviation; BMI: body mass index; Data were expressed as number, percentage, and mean ± SD.

^a^ Comparision between dominant hand position (DH) participants and non-dominant hand position (NH) participants

### Physiologic signs

Physiologic signs, including SBP, DBP, MAP, HR and SpO_2_ are shown in Table 2. After performing chest compressions, both non-dominant hand position and dominant hand position participants had a higher SBP and MAP, as well as a higher HR in both the constant hand position and switched hand position sessions (p<0.01, respectively). DBP was not statistically decreased after chest compression in the constant hand position session (p = 0.058 and 0.476, resp.) and the switched hand position session (p = 0.358 and 0.987, resp.) for both non-dominant hand position and dominant hand position participants. In addition, SpO_2_ was significantly decreased after chest compression in the two sessions for both non-dominant hand position and dominant hand position rescuers (p<0.001, resp.). However, there were no significant differences in the participants’ SBP, DBP, MAP, HR or SpO_2_ before or after chest compressions between the two sessions (p>0.05, respectively). The data of physiological signs of the dominant hand position and non-dominant hand position participants were merged for further analysis. However, no significant differences were observed (data not shown).

**Table 2:** Physiologic signs before and after chest compressions for participants (mean ± SD).

		CH session			SH session				
		pre-cc	post-cc	*p* value	pre-cc	post-cc	*p* value	*p* value ^a^	*p* value ^b^
NH participants (n = 119)	SBP (mmHg)	121.3 ± 13.1	130.2 ± 14.3	<0.001	122.8 ± 13.7	129.4 ± 15.2	<0.001	0.318	0.667
	DBP (mmHg)	73.1 ± 9.4	72.5 ± 8.4	0.058	73.3 ± 9.5	72.5 ± 8.6	0.358	0.862	0.328
	MAP (mmHg)	89.2 ± 9.5	91.0 ± 9.1	0.007	89.8 ± 9.7	91.5 ± 9.2	0.150	0.554	0.693
	HR (bpm)	89.4 ± 14.8	124.2 ± 18.5	<0.001	88.7 ±14.4	123.8 ± 19.7	<0.001	0.712	0.856
	SpO_2_ (%)	99.6 ± 0.8	98.9 ± 1.3	<0.001	99.6 ± 1.2	98.8 ± 1.4	<0.001	0.948	0.586
DH participants (n = 58)	SBP (mmHg)	125.5 ± 12.7	130.1 ± 13.2	0.043	122.8 ± 10.9	129.3 ± 11.6	<0.001	0.237	0.712
	DBP (mmHg)	73.8 ± 9.0	73.1 ± 8.2	0.476	72.8 ± 8.8	72.5 ± 8.8	0.987	0.553	0.813
	MAP (mmHg)	91.0 ± 8.8	92.1 ± 8.3	0.197	89.4 ± 8.6	91.6 ± 8.6	0.013	0.353	0.718
	HR (bpm)	88.5 ± 15.2	120.2 ± 21.2	<0.001	89.6 ± 15.1	117.8 ± 19.3	<0.001	0.689	0.446
	SpO_2_ (%)	99.5 ± 0.9	98.8 ± 1.4	<0.001	99.6 ± 0.8	98.9 ± 1.3	<0.001	0.813	0.548

CH: constant hand position; SH: switched hand position; DH: dominant hand position; NH: non-dominant hand position; pre-cc: before chest compressions; post-cc: after chest compressions; SBP: systolic blood pressure; DBP: diastolic blood pressure; MAP: mean arterial pressure; HR: heart rate; SpO_2_: peripheral capillary oxygen saturation.

^a^ comparison in pre-cc between two sessions;

^b^ comparison in post-cc between two sessions

### Chest compression and ventilation quality

Although all participants were asked to perform standard single adult CPR, the number of chest compressions in each cycle occasionally differed from that recommended by the guidelines. However, the total number of attempted chest compressions in the constant hand position and switched hand position sessions were similar for both non-dominant hand position and dominant hand position participants (p = 0.413 and 0.326, resp.) (Table 3). As shown in the Table 3, the total time of CPR was not statistically higher for the switched hand position session compared to the constant hand position session (p = 0.414 (NH) and 0.317 (DH), resp.). There was no significant difference in the hands-off time between cycles between the constant hand position and switched hand position sessions (p = 0.946 (NH) and 0.974 (DH), resp.). In addition, there were no significant differences between the constant hand position and switched hand position sessions in ventilation rate (p>0.05, resp.) or ventilation volume (p>0.05, resp.).

**Table 3:** Total number of chest compressions, hands off time, total CPR time and ventilations in two sessions (mean ± SD).

	Participants	CH session (n = 177)	SH session (n = 177)	Mean differences (95% CI)	*p* value
Total number of CC	NH	299.8 ± 26.2	301.7 ± 22.2	1.8 [-5.6, 9.3]	0.413
	DH	299.1 ± 21.7	301.5 ± 24.7	2.3 [-4.9, 9.6]	0.326
Hands-off time (s)	NH	24.1 ± 7.3	25.0 ± 6.1	0.81 [-6.8, 8.6]	0.946
	DH	23.5 ± 5.1	24.3 ± 5.7	0.80 [-6.5, 8.1]	0.974
Total time of CPR (s)	NH	214.3 ± 30.8	216.8 ± 32.7	2.5 [-4.9, 9.9]	0.414
	DH	217.3 ± 23.7	220.1 ± 29.0	2.8 [-10.2, 15.9]	0.317
VR (breath/min) ^a^	NH	3.5 ± 0.8	3.7 ± 0.8	0.2 [-0.3, 0.6]	0.976
	DH	3.7 ± 0.8	3.8 ± 0.4	0.2 [-0.3, 0.6]	0.897
VV (mL)	NH	681.6 ± 218.9	665.0 ± 217.4	16.7 [-49.9, 83.3]	0.398
	DH	650.0 ± 205.0	665.8 ± 181.2	15.0 [-42.4, 72.4]	0.420

CH: constant hand position; SH: switched hand position; CC: chest compressions; CPR: cardiopulmonary resuscitation; VR: ventilation rate;

^a^ ventilation was carried out with a bag-valve-mask (BVM). Ventilation rate was calculated as total number of ventilations to total CPR time (min). VV: ventilation volume; Data were expressed as mean ± SD

We also separately evaluated the chest compression rate and depth for the dominant hand position and non-dominant hand position participants (Table 4). For the dominant hand position participants, there were no significant differences in compression rate (SH vs. CH: 145.8 ± 14.3 vs. 147.8 ± 10.0 cpm, p = 0.857) or depth (40.9 ± 6.9 vs. 39.1 ± 10.0 mm, p = 0.227) between the two sessions. However, for the non-dominant hand position participants, chest compression depth was significantly higher in the switched hand position session than that in the constant hand position session (36.3 ± 8.1 vs. 39.3 ± 7.2 mm, p = 0.015), but no significant difference was found for compression rate between the switched hand position and constant hand position sessions (140.0 ± 13.5 vs. 143.2 ± 11.9 cpm, p = 0.057). These data were also merged for further analysis. Then, we found no significant differences in chest compression rate between the constant hand position and switched hand position sessions (p = 0.126). Chest compression depth was not statistically greater in the switched hand position session compared to the constant hand position session (p = 0.294)(data not shown). The percentages of complete recoil and correct hand placement on the chest were also recorded to evaluate chest compression quality (Table 4). The percentage of complete chest compressions in the constant hand position session was not statistically higher than that in the switched hand position session for neither dominant hand position rescuers (52.8 ± 6.2% vs. 51.8 ± 5.7%, p = 0.775) nor non-dominant hand position rescuers (54.3 ± 7.6% vs. 52.3 ± 4.3%, p = 0.247). In addition, the percentage of correct hand placement on the chest was similar between the constant hand position and switched hand position sessions for both dominant hand position rescuers (89.8 ± 7.8% vs. 90.8 ± 8.6%, p = 0.649) and non-dominant hand position rescuers (89.8 ± 9.2% vs. 91.7 ± 10.4%, p = 0.206)

**Table 4:** Chest compression rate, depth, recoil and hand placement in two sessions (mean ± SD).

	Participants	CH session (n = 177)	SH session (n = 177)	Mean differences (95% CI)	p value
Compression rate (cpm)	NH	143.2 ± 11.9	140.0 ± 13.5	-3.2 [-7.2, 0.8]	0.057
	DH	147.8 ± 14.3	145.8 ± 10.0	-2.0 [-9.7, 5.7]	0.857
Compression depth (mm)	NH	36.3 ± 8.1	39.3 ± 7.2	3.0 [1.0, 5.0]	0.015
	DH	39.1 ± 10.0	40.9 ± 6.9	1.8 [-0.3, 3.9]	0.227
Compression recoil (%)	NH	54.3 ± 7.6	52.3 ± 4.3	-1.7 [-5.5, 2.1]	0.247
	DH	52.8 ± 6.2	51.8 ± 5.7	-1.0 [-4.5, 2.5]	0.775
Appropriate Hand placement (%)	NH	89.8 ± 9.2	91.7 ± 10.4	-1.8 [-4.4, 0.8]	0.206
	DH	89.8 ± 7.8	90.8 ± 8.6	-1.0 [-2.6, 0.6]	0.649

CH: constant hand position; SH: switched hand position; DH: dominant hand position; NH: non-dominant hand position; Data were expressed as mean ± SD

### Fatigue

**Fig 2:**
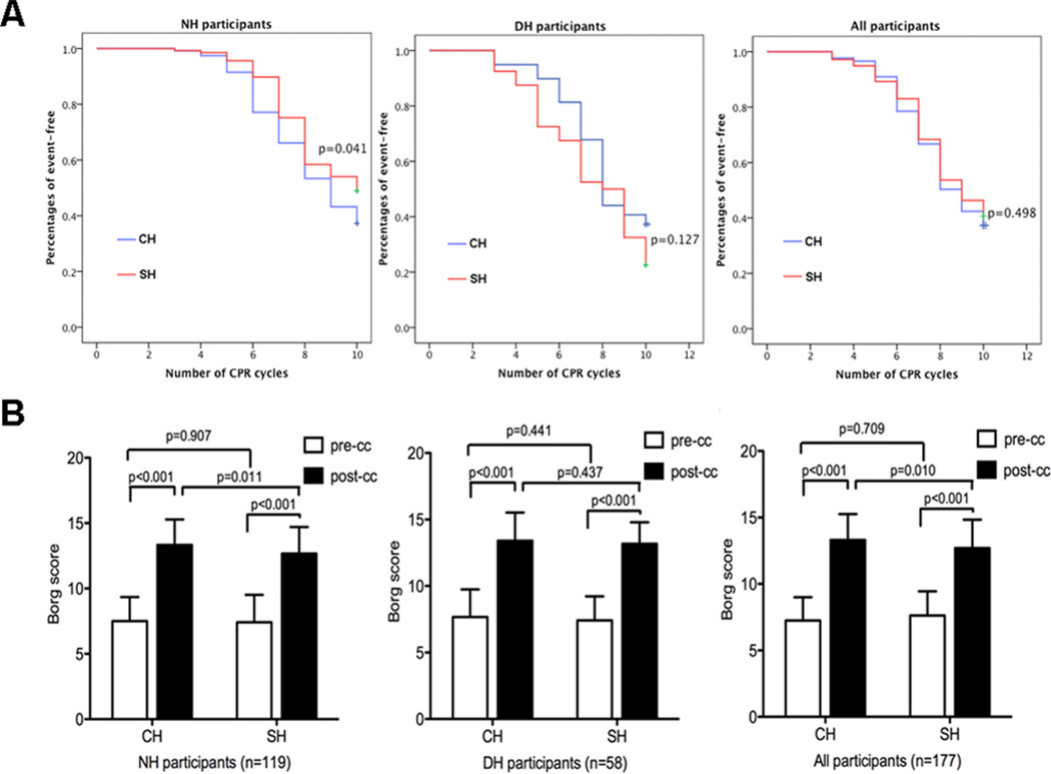
Fatigue appearance (A) and Borg score (B). “Event-free” means “no fatigue appearance”. CH: constant hand position; SH: switched hand position; NH: non-dominant hand position; DH: dominant hand position. pre-cc: before chest compressions; post-cc: after chest compressions.

In this study, we recorded physiologic signs, Borg score and the cycle number of fatigue appearance to evaluate the participants’ fatigue. Participants achieved a significantly higher Borg score after chest compressions in both the constant hand position (post CH vs. pre CH: 13.4 ± 2.0 vs. 7.4 ± 1.8, p<0.001, resp.) and switched hand position (post SH vs. pre SH: 13.11 ± 1.78 vs.7.45 ± 2.03, p<0.001, resp.) sessions (Fig 2) compared with pre-compression. Because all participants were asked to perform a standard single adult CPR with hands-off time between cycles, we recorded the cycle number of fatigue appearance instead of the exact time of fatigue appearance. We separately evaluated the data of non-dominant hand position and dominant hand position participants, and no significant differences were found for Borg score (13.41 ± 2.11 vs.13.17 ± 1.62,p = 0.437) or cycle number (p = 0.127) of fatigue appearance after chest compressions for dominant hand position participants between two sessions. However, for the non-dominant hand position participants, the Borg score was significantly lower after chest compressions in the switched hand position session than in the constant hand position session (12.67 ± 2.03 vs. 13.33 ± 1.95, p = 0.011). Moreover, the appearance of fatigue in the switched hand position session was later than in the constant hand position session (p = 0.041). Over the whole study population, the CPR cycle number of fatigue appearance was similar (p = 0.498); however, the Borg score was significantly higher after chest compressions in the constant hand position session than in the switched hand position session (14 ± 2 vs. 13 ± 2, *p* = 0.010).

## Discussion

We performed a manikin study to investigate the effects of up-down hand position switch during single adult CPR on rescuer fatigue and CPR quality. We found that up-down hand position switch during CPR decreases Borg score, but it does not improve CPR quality in regards to chest compressions and ventilations. However, different findings were observed when the data for the dominant hand position and non-dominant hand position rescuers were considered separately. Hand position switch significantly delayed rescuer fatigue and increased chest compression depth for the non-dominant hand position rescuers but not for the dominant hand position rescuers.

This study included novice rescuers because the results of our previous manikin study suggested that dominant hand position could delay the fatigue of novice rescuers and improve ECC quality [5]. The findings of our previous study were in contrast to the results of *Nikandish et al*., who showed that dominant hand placement on the sternum during CPR failed to increase ECC quality [4]. There are several possible explanations for this difference between these two studies. In contrast to *Nikandish et al*., we asked the rescuers to perform chest compressions with ventilations at a ratio of 30:2 instead of performing hands-only CPR. Although the survival rates for patients who experienced CA of cardiac etiology are similar for hands-only CPR and CPR with both compressions and ventilations, the 2010 AHA guidelines recommend that trained rescuers perform both chest compressions and ventilations [10]. In addition, the sample size in our previous study was much greater than that in the study performed by *Nikandish et al*. (220 vs. 59).

In the present study, approximately 67% (119/177) of the participants preferred placing the non-dominant hand in contact with the sternum during chest compressions, which was in line with our previous findings. For non-dominant hand position rescuers, we hypothesized that hand position switch during CPR would decrease fatigue and increase chest compression quality, and our results showed that hand position switch increased compression depth and decreased compression rate for dominant hand position rescuers. In the current guidelines, chest compressions should be initiated using the C-A-B sequence [11]. However, it is common for rescuers to perform CPR with an excessively higher compression rate than that recommended by the current AHA guidelines [12], and the push-fast technique tends to increase rescuer fatigue, which can result in an early decay of CPR quality [12]. *Monsieurs et al*. also reported that an excessively high chest compression rate could result in insufficient compression depth [13]. In the present study, we observed that both dominant hand position and non-dominant hand position rescuers performed CPR with an excessive compression rate of approximately 150 cpm. However, the chest compression rate was not statistically decreased with the up-down hand position switch. Thus, lowering chest compression rate may result in a decrease in the loss of compression depth. Finally, the up-down hand position switch during CPR increased the compression depth of non-dominant hand position rescuers, but not dominant hand position rescuers. We separately analyzed the characteristics of both the non-dominant hand position and dominant hand position rescuers. The only difference between the dominant hand position and non-dominant hand position rescuers was in weight; dominant hand position rescuers had significantly greater weight than the non-dominant hand position rescuers. However, a manikin study by *Ødegaard et al*. showed that compression depth does not depend on rescuer sex, height or weight [14].

*Yang et al*. reported that following the 2010 American Heart Association guidelines improved the quality of chest compressions but increased rescuer fatigue as compared with following the 2005 AHA guidelines [15]. In that study, each rescuer performed hands-only CPR for only 8 minutes. However, chest compressions might be performed much longer during real life CPR, and rescuer fatigue can increase over the time. It is possible that severe fatigue can decrease the quality of chest compressions at the later stage of CPR. In fact, a manikin study based on the 2010 European Resuscitation Council (ERC) guidelines showed that fatigue affects chest compression delivery within the second minute of CPR [16]. Therefore, under the current guidelines, rescuer fatigue has already become a concern, as it may impact the quality of CPR. Many studies have recommended alternating rescuers every 2 min or even more frequently [17]; however, a sufficient number of rescuers to allow for this are not always present in real life. In fact, the majority of out-of-hospital cardiac arrests (OHCA) occur at home, and the response time of emergency medical services is much greater than 5 min [18]. Therefore, several studies aimed to use different tools, such as the CPR PRO (CPRO) device [19], step tool [20], and metronome [21] to reduce the fatigue of rescuers and improve the quality of CPR. In our study, fatigue was delayed by switching hand position without the use of any other tools. Thus, this modification to hand positioning during CPR could be a simple and effective method for reducing fatigue and improving CPR quality.

Several indicators, including heart rate (HR) [19], respiratory rate (RR) [22], blood pressure [21], venous serum lactate level [23] and Borg score [23] have been used to evaluate the rescuer fatigue in manikin studies. However, we did not find any gold standard indicators for the measurement of rescuer fatigue; thus, the accuracy of indicators of rescuer fatigue remains unclear. In this study, we recorded physiologic signs, including blood pressure, heart rate, cycle number of fatigue appearance and Borg score. We only observed differences in cycle number of fatigue appearance and Borg score when dominant hand position rescuers used the hand position switch.

## Limitations

There are several limitations to this study. First, this study was performed with manikins in a simulated setting; thus, the results may not reflect the clinical setting. Secondly, participants in the switched hand position session switched their hand position every two CPR cycles. The frequency of hand position switch used in this study was chosen randomly. It is possible that switching hand position at other frequencies may have different results. Thirdly, we asked participants to perform a standard single adult CPR with ventilations; however, the data were not analyzed cycle by cycle. It is unclear whether differences existed between the cycles. Finally, we enrolled only novice rescuers in this study; thus, the effects of hand position switch on CPR quality among professional healthcare providers should be investigated in future studies.

## Conclusion

Following 2010 AHA CPR guidelines, the up-down hand position switch during CPR may increase the external chest compression depth without altering the compression rate and ventilation quality, and may delay the fatigue of novice non-dominant hand position rescuers but not dominant hand position rescuers.
